# Twisted Gut, Stricken Liver: A Case of Ischemic Hepatitis From Caecal Volvulus

**DOI:** 10.7759/cureus.73002

**Published:** 2024-11-04

**Authors:** Amarah Shaikh, Jada Saunders, Isran Shah, Khalid Khalifa

**Affiliations:** 1 General Surgery, Kingston Hospital NHS Foundation Trust, London, GBR

**Keywords:** caecal volvulus, colonic volvulus, hypoxic hepatitis, ischemic hepatitis, laparotomy

## Abstract

Caecal volvulus, accounting for a significant proportion of colonic volvulus cases, involves the axial twisting of the mobile caecum. While ischemic hepatitis is conventionally associated with specific etiologies, reports linking it to caecal volvulus are scarce.

This case report describes a noteworthy presentation of ischemic hepatitis triggered by caecal volvulus in an elderly woman of 80 years who presented with acute epigastric pain and laboratory evidence of acute liver injury, prompting imaging studies that unveiled features suggestive of caecal volvulus.

## Introduction

Caecal volvulus, a distinctive manifestation accounting for approximately 25-40% of all cases of colonic volvulus, is typified by the axial twisting of the caecum. This pathological condition tends to manifest predominantly in individuals with a notable caecum [1]. Approximately 25% of the cardiac output is directed to hepatic blood flow, supplied through two vascular systems: the portal vein system, responsible for roughly two-thirds of the blood flow, and the arterial hepatic system, contributing to about one-third. Consequently, the liver is effectively safeguarded against ischemia, primarily attributed to the presence of this dual circulatory system [2]. This report contributes a distinctive case study, shedding light on the rare occurrence of ischemic hepatitis induced by caecal volvulus, compressing onto the porta hepatis, in an elderly patient. The ensuing exploration of this case not only enriches our understanding of the intricacies surrounding caecal volvulus but also highlights the potential implications for hepatic vascular compromise in the context of this colonic pathology.

## Case presentation

An 80-year-old female, with a medical history of hypertension, a non-functioning kidney of unknown cause, and lymphedema, presented to the emergency department with sudden severe epigastric pain, nausea, and constipation. Physical examination revealed dehydration, tachycardia (HR 103 bpm), and epigastric tenderness with generalized guarding. Laboratory results indicated liver injury, with elevated bilirubin (24 µmol/L), serum alanine aminotransferase (1400 U/L), alkaline phosphatase (202 U/L), and lactate (7.1 mmol/L), alongside a white blood cell count of 13.6 x 10^9/L and CRP of 4.0 mg/L. A CT scan identified a large thrombus in the main portal vein, involving the right and left portal veins and a likely non-occlusive thrombus in the superior mesenteric vein. Diffuse thickening of the small and large bowel, fat stranding, and congestion in the mesentery raised concerns for bowel ischemia. Further findings included caecal volvulus, chronic pelvic ureteric junction obstruction of the right kidney, and a hypodense left lobe of the liver. Despite initial resuscitation, her laboratory results worsened, with aspartate aminotransferase (AST) increasing to 12,049 U/L, alkaline phosphatase (ALP) to 253 U/L, bilirubin to 71 µmol/L, further increases in C-reactive protein and white cell count, and a deranged coagulation profile.

**Figure 1:**
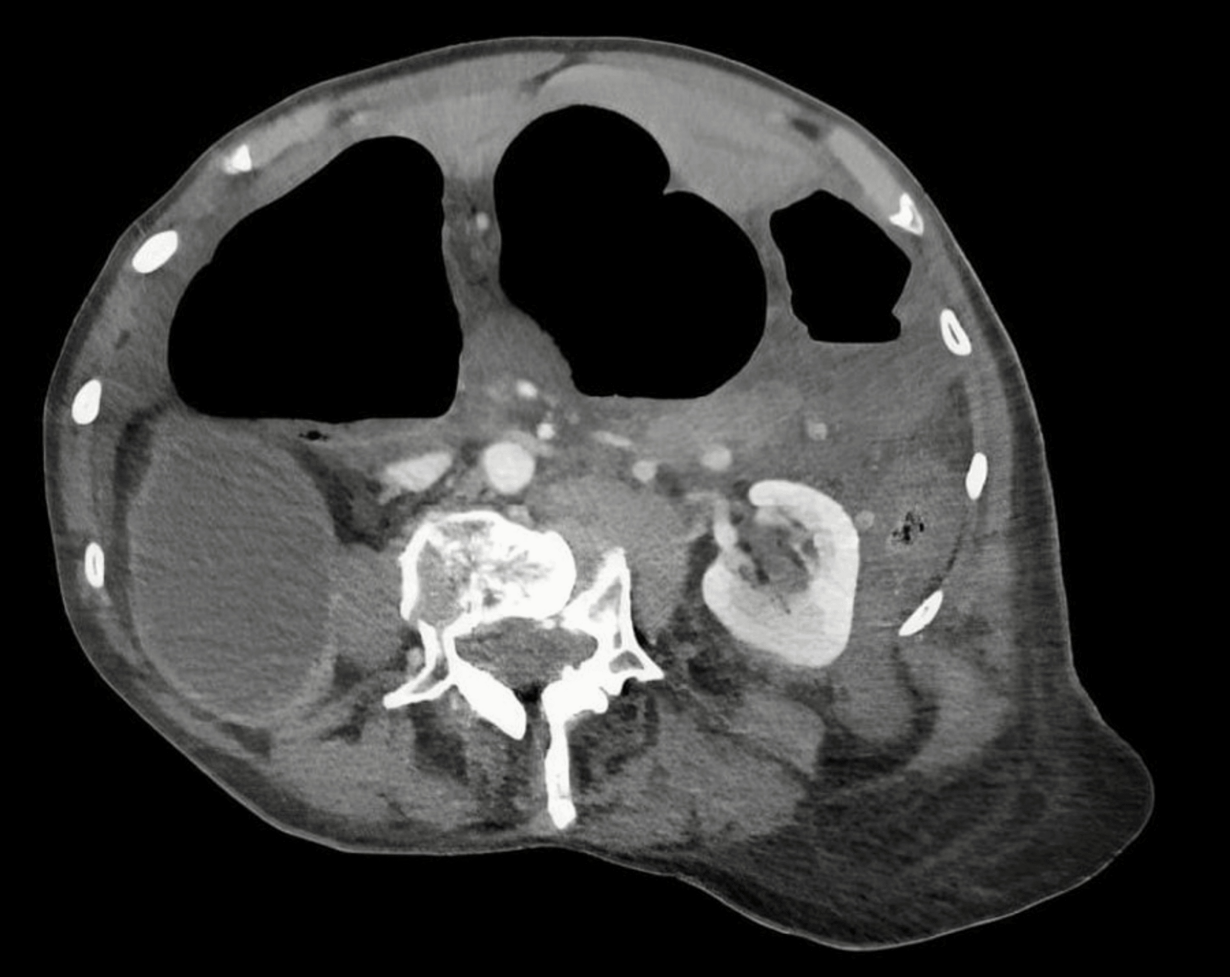
CT image suggesting volvulus

**Figure 2:**
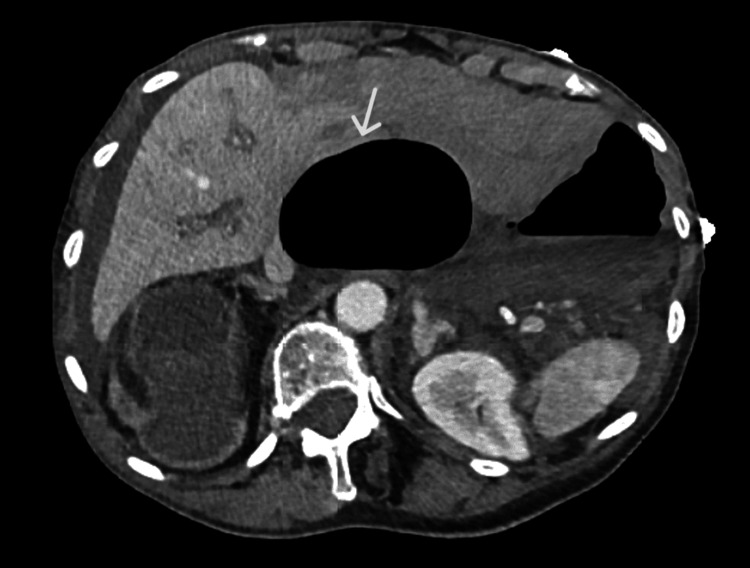
Volvulus compressing the porta hepatis

Due to the patient's deteriorating hemodynamic and biochemical status, combined with the concerning CT scan findings (Figure 1), an emergency exploratory laparotomy was performed. This revealed an omental band forming an internal hernia, trapping the caecum and causing a type 2 caecal volvulus. A grossly dilated necrotic caecum was found pressing on the porta hepatis, likely leading to ischemic hepatitis (Figure 2). The necrotic caecum was deflated via a purse-string controlled enterotomy without spillage. The omental band was divided, and a double-barrel ileostomy was formed. Postoperatively, the patient was transferred to the ICU for management of ischemic hepatitis (shock liver), probable portal vein thrombosis, and multi-organ failure, requiring cardiac, renal, and respiratory support.

The patient exhibited severe acute kidney injury, coagulopathy, and hypoalbuminemia. Repeat imaging to evaluate the portal vein thrombosis was inconclusive but revealed bilateral pleural effusions, lung atelectasis, and a heterogeneous left lobe of the liver. With no evidence of splenomegaly or ascites to suggest cirrhotic changes and a negative liver screen, it was concluded that the liver injury was due to hypoperfusion caused by the pressure effect from the caecal volvulus. The patient's overall prognosis was guarded, and she was managed for ischemic hepatitis with N-acetylcysteine, inotropic support, and furosemide infusion for AKI. She required ongoing intensive care due to persistent challenges, including coagulopathy and respiratory complications.

Gradually, her condition began to improve. Her stoma started functioning on the fourth post-operative day but was noted to be prolapsed with slight ischemic changes, though it remained functional. She was started on nasogastric feeding and was extubated on the eleventh post-operative day. Her liver function tests showed gradual improvement, normalizing by the fourteenth-day post-surgery. She continued to recover and was transferred out of the ICU after 16 days. Due to the prolapsing stoma, which required multiple manual reductions, she underwent a stoma refashioning one month after the initial surgery. Despite a prolonged hospital stay, she was eventually discharged in a haemodynamically and clinically stable condition. On telephonic follow-up, she reported to be doing well and coping with the stoma.

## Discussion

Ischemic hepatitis is characterized by a swift and temporary surge in plasma aminotransferase levels, typically observed in conditions such as heart failure, circulatory or septic shock, and respiratory failure [2]. The histological hallmark involves hepatocellular necrosis in the centrilobular zone, termed hepatic centrilobular necrosis. The terms "hypoxic hepatitis" (HH) or "shock liver" are employed in literature, emphasizing multifactorial pathophysiologic factors leading to hypoxia and subsequent alterations [3]. Prognosis varies, ranging from complete regression to short-term mortality, contingent on the underlying aetiology and haemodynamic severity. Management focuses on correcting hypoperfusion and liver congestion, facilitating the regression of liver enzyme levels. AST levels rapidly decrease within 24-48 hours while ALT and lactate dehydrogenase (LDH) levels gradually return to normal values over one to two weeks [2].

Volvulus is defined as a twisted loop of the intestinal bowel and associated mesentery around a fixed point at its base. Three types of caecal volvulus are outlined in the literature: Type 1 involves clockwise axial twisting, Type 2 arises from the twisting of the cecum and terminal ileum with displacement to an ectopic location (usually left upper quadrant), usually in a counterclockwise manner. Type 3, known as caecal bascule, entails upward folding without axial twisting [4]. In this case, It was Type 2 volvulus causing pressure on the porta hepatis and in the right upper quadrant, which is unusual. Upon presentation, our patient exhibited neither hypoxia nor multi-organ failure, which might typically explain ischemic hepatitis. Instead, it was concluded that the condition was likely induced by the pressure effect of caecal volvulus, with additional factors implicated by systemic hypoperfusion indicated by the patient's dehydrated appearance and tachycardia upon admission. While intestinal volvulus leading to mesenteric ischemia might result in ischemic colitis, observing ischemic hepatitis due to caecal volvulus in an individual with an otherwise normal liver is uncommon. Nonetheless, a reported case highlights ischemic hepatitis resulting from sudden and temporary portal vein occlusion caused by mesenteric volvulus in a patient with chronic hypoxemia due to underlying chronic obstructive pulmonary disease [5].

## Conclusions

In summary, this case study emphasizes the infrequent incidence of ischemic hepatitis resulting from caecal volvulus. The patient's initial presentation and subsequent diagnostic revelations unveiled a distinct manifestation of type 2 caecal volvulus, exerting pressure on the porta hepatis and leading to ischemic hepatitis. The thorough examination of this case not only enhances our comprehension of the complexities related to caecal volvulus but also underscores the potential repercussions for hepatic vascular compromise within the realm of this colonic pathology. Although the patient stabilized and exhibited improvement in liver function tests, her demise from an unidentified cause in the community three months post-discharge is regrettable. Despite this unfortunate outcome, the case report provides valuable insights into the intricate clinical aspects and management challenges linked to ischemic hepatitis induced by caecal volvulus. It emphasizes the crucial necessity for increased awareness and consideration of this rare complication in similar clinical scenarios. Caecal volvulus, although infrequent, should be considered in the spectrum of potential triggers for this condition, emphasizing the need for comprehensive evaluations and tailored interventions.
